# The Poison in Cheese

**Published:** 1886-02

**Authors:** 


### THE POISON IN CHEESE.

It is well known that severe cases of poison have occurred from the eating of cheese. It has frequently happened in this country and cases have been known in Germany. It is a common expression among Americans who are effected by it that they have partaken of sick cheese. It is now well known that this effect is sometimes found in cheese of large dairies, although at one time, it was common to attribute it to cheese made on farms, in small quantities. The cheese made at a large factory in Ohio and Michigan convinced people that there were other causes than mere small makers, or inefficient ones for the existence of the poison. After long and patient investigation Prof. Vaughn of Michigan has given to the Board of Health of that State the valuable results of his investigations. Cats and dogs are not effected by it although they invariably choose the good cheese from the poor, when both are given to them to choose from. Possibly if a person tasted a cheese knowing that it was poisonous he might detect a sharpness of taste which would not ordinarily be noticed. But there is no certain means, aside from a chemical examination, by which a poisonous cheese can be distinguished from a wholelsome one. The most trustworthy, ready method of examination is to press a slip of blue l.tmus paper against a freshly-cut surface of the cheese. If the paper is reddened instantly and intensely the cheese may be regarded with suspicion. When treated in this way any green cheese will redden the litmus paper, but ordinarily the reddening will be produced slowly and will be slight. If the piece of cheese be dry it should be rubbed up with an equal volume of water, and the paper should be dipped in the water. Dr. Vaughan thinks that grocerymen should apply this test to every fresh cheese.

After a long and determined hunt the Professor succeeded in isolating the poison, which will now pass into chemical science under the name of tyrotoxicon. It is found to be a product of imperfect putrefaction in the cheese, and it occurs in the manufacturing vat, for the curd itself has been known to poison persons. Tyrotoxicon appears in the form of needle-shaped crystals, which are freely soluble in water. The smallest visible fragments of a crystal placed upon the tongue caused a sharp, stinging pain and in a few minutes dryness and constriction of the throat. A slightly larger amount produced vomiting, nausea and diarrhœa. The isolated poison has a sharp pungent odor, but in the cheese the taste and odor of the poison are both modified beyond recognition. The poison is volatile, and even posionous cheese may be eaten after it is cooked.

The symptoms observed in cheese poisoning are similar to those caused by tyrotoxicon, with the addition of head-ache, double vision, and marked nervous prostration. In rare instances the sufferer dies of collapse.

